# Natakalim improves post-infarction left ventricular remodeling by restoring the coordinated balance between endothelial function and cardiac hypertrophy

**DOI:** 10.3892/ijmm.2014.1931

**Published:** 2014-09-12

**Authors:** HONG-MIN ZHOU, MING-LI ZHONG, YAN-FANG ZHANG, WEN-YU CUI, CHAO-LIANG LONG, HAI WANG

**Affiliations:** 1 Cardiovascular Drug Research Center, Institute of Health and Environmental Medicine, Academy of Military Medical Sciences, Beijing 100850, P.R. China; 2 Cardiovascular Drug Research Center, Thadweik Academy of Medicine, Beijing 100039, P.R. China

**Keywords:** natakalim, myocardial infarction, left ventricular remodeling, cardiac hypertrophy, congestive heart failure, endothelial function

## Abstract

Endothelial dysfunction can lead to congestive heart failure and the activation of endothelial ATP-sensitive potassium (K_ATP_) channels may contribute to endothelial protection. Therefore, the present study was carried out to investigate the hypothesis that natakalim, a novel K_ATP_ channel opener, ameliorates post-infarction left ventricular remodeling and failure by correcting endothelial dysfunction. The effects of myocardial infarction were assessed 8 weeks following left anterior descending coronary artery occlusion in male Wistar rats. Depressed blood pressure, cardiac dysfunction, evidence of left ventricular remodeling and congestive heart failure were observed in the rats with myocardial infarction. Treatment with natakalim at daily oral doses of 1, 3 or 9 mg/kg/day for 8 weeks prevented these changes. Natakalim also prevented the progression to cardiac failure, which was demonstrated by the increase in right ventricular weight/body weight (RVW/BW) and relative lung weight, signs of cardiac dysfunction, as well as the overexpression of atrial and brain natriuretic peptide mRNAs. Our results also demonstrated that natakalim enhanced the downregulation of endothelium-derived nitric oxide, attenuated the upregulation of inducible nitric oxide synthase-derived nitric oxide (NO), inhibited the upregulated endothelin system and corrected the imbalance between prostacyclin and thromboxane A_2_. Overall, our findings suggest that natakalim prevents post-infarction hypertrophy and cardiac failure by restoring the coordinated balance between endothelial function and cardiac hypertrophy.

## Introduction

Left ventricular remodeling (LVR), including cardiac hypertrophy, myocardial fibrosis and changes in left ventricular shape, is an adaptation to preserve cardiac output in a number of pathological conditions, such as hypertension and myocardial infarction (MI). However, deteriorated LVR may eventually lead to congestive heart failure (CHF) (1,2). There are several factors involved in the progression of LVR to CHF; however, endothelial function has received increasing attention. The vascular endothelium is widely distributed, and it plays a very important role in the pathophysiological process of cardiovascular diseases. The important roles of the coronary endothelium include not only the control of blood flow, but also the regulation of cardiomyocyte structure and function through autocrine or paracrine signaling via many vasoactive substances (3). Additionally, previous studies have demonstrated that deteriorative left ventricular dysfunction is associated with coronary endothelial dysfunction (4–6). Moreover, in the study by Shiojima *et al* (7), it was suggested that the imbalance between endothelial function and the adaptation of cardiac hypertrophy causes the transition from LVR to failure. Therefore, correcting coronary endothelial dysfunction in the residual myocardium is a very important therapeutic strategy for the clinical treatment of patients with post-infarction CHF.

The ATP-sensitive potassium channel (K_ATP_) is widely distributed in the cardiovascular system and plays important roles. K_ATP_ consists of inward rectifier potassium channel subunits (Kir6s) and regulative sulfonylurea receptors (SURs) (8); the SUR2B/Kir6.1 subtype is mainly found in the vascular endothelium (9). It has previously been reported that the protein expression of Kir6.1 is increased in the ischemic myocardium and that the dysfunction of Kir6.1/SUR2 is involved in cardiac hypertrophy and heart failure (1). Our laboratory has previously reported that the activation of the SUR2B/Kir6.1 subtype prevents cardiac hypertrophy and the progression from hypertrophy to failure induced by pressure overload (11,12). Therefore, it can by hypothesized that correcting endothelial dysfunction by activating Kir6.1/SUR2B channels in the endothelium may prevent post-infarction CHF. Natakalim, a novel K_ATP_ channel opener, can reverse vascular endothelial cell dysfunction caused by homocysteine and hypoxia and prevent the progression of cardiac hypertrophy to failure induced by pressure overload by selectively opening the SUR2B/Kir6.1 channel subtype (12–14). Based on this evidence, we hypothesized that natakalim may improve post-infarction LVR and CHF by protecting against coronary endothelial dysfunction in the residual myocardium.

The progression from occlusion of the left anterior descending coronary artery (LAD) to post-infarction CHF in rats is similar to what occurs when a patient survives a large MI but subsequently develops heart failure (15,16). However, few experimental studies have attempted to clarify the effects of activating the SUR2B/Kir6.1 channel subtype on LVR and CHF using this model. Therefore, to the bets of our knowledge, for the first time, we evaluated the pharmacological characteristics and experimental therapeutic effects of natakalim on LVR and CHF in a rat model of ligation of the LAD. We also explored whether natakalim improves post-infarction CHF by restoring the coordinated balance between endothelial function and cardiac hypertrophy in the residual myocardium.

## Materials and methods

### Reagents

Natakalim was synthesized at the Thadweik Academy of Medicine (Beijing, China). All other chemicals and materials were obtained from local commercial sources.

### Modle of MI and study protocol

All procedures were performed in accordance with the protocol outlined in the Guide for the Care and Use of Laboratory Animals published by the US National Institutes of Health (NIH publication no. 85-23, revised in 1996) and approved by the local animal care and use committee. Experiments were performed using healthy adult male Wistar rats (weight range, 200±30 g), and the animals were divided into 6 groups (n=9–11) as follows: the sham-operated group, which included animals that underwent a similar procedure without left coronary artery ligation and were treated with distilled water; the model group, which included rats with MI induced by LAD ligation that were treated with distilled water; rats with MI that were treated with 1, 3 or 9 mg/kg/day of natakalim; and rats with MI treated with a positive control drug (lisinopril) at 15 mg/kg/day. No significant difference was found among all the experimental groups as regards age or body weight (BW) prior to surgery.

The rat model of MI was implemented as previously described (15), with slight modifications. First, following anesthesia with pentobarbital (40 mg/kg, intraperitoneal injection), the trachea of the rat was incised and cannulated to allow artificial ventilation by a volume-constant rodent ventilator under sterile conditions. Second, a left thoracotomy was performed at the fourth intercostal space, and the ribs were opened with a hemostatic clamp to expose the heart. After opening the pericardium, the left coronary artery was ligated using a 7.0 silk suture close to the artery origin (2 mm below). Subsequently, the rib cage, muscle layer and skin were closed separately. Successful occlusion was immediately confirmed by ECG (ST-elevation) and the observation of an arising pale ischemic zone with weak activity. The drugs were dissolved in normal saline and administered orally via a gastric tube once a day for 8 weeks from the third post-operative day. At the end of the 8 weeks, the rats were sacrificed for histological evaluation.

### Hemodynamics and cardiac remodeling index

On day 56, following anesthesia with a mixture of xylazine (5 mg/kg) and pentobarbital (15 mg/kg, intraperitoneal injection), the right carotid was cannulated with a polyethylene catheter (outer diameter of 0.96 mm) to measure the carotid arterial pressure. Subsequently, a catheter was inserted along the right carotid artery into the left ventricle, and the signals were recorded on an 8-channel direct-writing oscillograph (RM-6000; Nihon Kohden Corporation, Tokyo, Japan) and digitally sampled (1 kHz) on a personal computer equipped with an analog-to-digital interface (SMUP-PC bioanalysis system; Jialong Instrument, Shanghai, China). The heart rate (HR), systolic blood pressure (SBP), diastolic blood pressure (DBP), left ventricular systolic pressure (LVSP), left ventricular end diastolic pressure (LVEDP), maximal rate of left ventricular systolic and diastolic pressure (±d*p*/d*t*_max_), physiological velocity of contractile element shorting (Vpm) and maximal velocity of contractile element shorting (Vmax) were recorded.

Thereafter, blood samples were collected and the animals were sacrificed by exsanguination. The still-beating heart was exposed after thoracic cavity opening. The hearts and lungs were rapidly removed, rinsed in ice-cold 0.9% NaCl solution, blotted and weighed. The heart weight (HW)/BW ratio (HW/BW) was calculated by dividing the HW by the BW; the left ventricles (including interventricular septum) and right ventricular free walls were then collected separately and weighed. The left ventricular weight (LVW)/BW (LVW/BW) ratio, the right ventricular weight (RVW)/BW ratio (RVW/BW) and the lung weight (LW)/BW ratio (LW/BW) were calculated.

### Histological analysis and transmission electron microscopy

The hearts were immersion-fixed in neutral 10% buffered formalin and paraffin sections (5 μm) were cut. The myocyte cross-sectional area and myocardial fibrosis were quantitatively analyzed using NIH Image 1.61 software (National Institutes of Health Service Branch) in digitized microscopic images. For measurement of the cross-sectional area, 100 cells (per animal, hematoxylin and eosin-stained, ×400 magnification) from the left ventricular mid-lateral free wall (including epicardial and endocardial portions) were randomly selected and analyzed. Interstitial myocardial fibrosis in the tissue sections was quantitatively analyzed by morphometry (in Masson’s trichrome-stained sections, ×100 magnification). The collagen in the myocardial interstitial spaces was visualized and the whole areas of the sections were scanned. The total interstitial fibrosis index was defined as the sum of the total area of collagen in the entire visual field divided by the sum of total connective tissue area plus the myocardial area in the entire visual field. All the images were digitized and transformed into binary images, and the areas occupied by collagen were calculated by an automatic area-quantification program using NIH Image software.

Myocardial samples were routinely fixed in 2.5% glutaraldehyde in 0.1 M phosphate buffer (pH 7.3) and then fixed in buffered 1% osmium tetroxide for 1 h. The samples were then dehydrated in a series of ethanol (50% ethanol, 5 min; 75% ethanol, 5 min; 90% ethanol, 5 min; and 100% ethanol, 5 min) and embedded in Epon 812. Thin sections were stained with uranyl acetate and lead citrate and examined under a Philips CM-120 electron microscope (Royal Dutch Philips Electronics Ltd, Amsterdam, The Netherlands). Muscle fiber and mitochondrial abnormalities were evaluated.

### Measurement of nitric oxide (NO), endothelin (ET)-1, prostacyclin [prostaglandin I2 (PGI_2_), thromboxane A_2_ (TXA_2_) and hydroxyproline levels

Blood was obtained from the right carotid artery with a polyethylene catheter at the time of sacrifice. The level of NO in serum was measured using the NO Detection kit (Nanjing Jiancheng Bioengineering Institute, Nanjing, China) according to the manufacturer’s instructions. ET-1, PGI_2_ and TXA_2_ levels were measured using commercial radioimmunoassay kits (Eastern Asia Radioimmunity Research Institute, Beijing, China) according to the manufacturer’s instructions. The hydroxyproline content in the hearts was measured using a commercial kit (Nanjing Jiancheng Bioengineering Institute) according to the manufacturer’s instructions.

### Immunohistochemistry

Immunohistochemical staining was performed using the UltraSensitive™ S-P kit (Wuhan Boster Bio-engineering Co, Ltd, Wuhan, China) according to the manufacturer’s instructions. Anti-endothelin receptor A and B (Sigma-Aldrich) were used as primary antibodies at a 1:100 dilution. Images from the entire sections were acquired using a digital camera system (Leica DFC295; Leica, Solms, Germany). Confocal images were then transferred to a personal computer using the NIH Image software package for image analysis, as previously reported (11) and with minor modifications.

### Western blot analysis

Protein was isolated from frozen non-infarcted left ventricular tissue using cell lysis buffer for western blotting (Beytime Institute of Biotechnology, Haimen, China) in accordance with the manufacturer’s instructions. Equal amounts of protein extracts were separated by 8% SDS-PAGE gel and then transferred onto PVDF membranes using a Bio-Rad transfer blotting system (Bio-Rad Laboratories, Hercules, CA, USA). The membranes were incubated with antibodies against endothelial NO synthase (eNOS) and inducible NO synthase (iNOS) (Abcam, Cambridge, MA, USA). Proteins were visualized using an enhanced chemiluminescence detection system (ECL; Cell Signaling Technology Inc). Anti-GAPDH antibody (BioEasy) was used to control for equal protein loading.

### Quantitative reverse transcription PCR (RT-qPCR)

Total RNA was extracted from the cardiac tissue of rats using TRIzol reagent according to the manufacturer’s instructions (Invitrogen, Carlsbad, CA, USA) and converted to cDNA using the Quantscript RT kit (Tiangen Biotech Co., Ltd., Beijing, China). Quantitative PCR was performed using the Real-Time PCR iCycler iQ5 System (Bio-Rad Laboratories) and SYBR-Green PCR Master Mix (Toyobo, Osaka, Japan) in accordance to the manufacturer’s instructions. The expression levels of GAPDH were used for sample normalization. Primers for rat atrial natriuretic peptide (ANP), brain natriuretic peptide (BNP) and GAPDH were as follows: ANP sense, 5′-ATT TCA AGA ACC TGC TAG ACC-3′ and antisense, 5′-CAA TCC TGT CAA TCC TAC CC-3′, 300 bp; BNP sense, 5′-GGA CCA AGG CCC TAC AAA AGA ACT-3′ and antisense, 5′-ACA ACC TCA GCC CGTCAC AGC-3′, 176 bp; GAPDH sense, 5′-AAA CCT CCA AGT ATG ATG AC-3′ and antisense, 5′-TTG TCA TAC CAG GAA ATG AGC-3′, 197 bp. The reactions were incubated at 95°C for 1 min, followed by 40 cycles at 95°C for 15 sec, 66.5°C for 15 sec and 72°C for 45 sec.

### Statistical analysis

In this stuyd, the results are expressed as the means ± SD. Statistical analysis of the data was performed using one-way ANOVA with the SPSS 13 software program. Probability values (P-values) ≤0.05 were considered to indicate statistically significant differences.

## Results

### Hemodynamic effects of natakalim

Carotid arterial pressure and cardiac function were measured 8 weeks following LAD occlusion. As shown in Table I, compared with the sham-operated rats, the mean arterial blood pressure (MABP) of the right carotid aorta was significantly reduced in the rats with MI. This was prevented by treatment with natakalim at daily oral doses of 1, 3 or 9 mg/kg for 8 weeks. The *in vivo* left ventricular function measurements for all the groups are shown in Table I. Systolic cardiac parameters, including LVSP, +d*p*/d*t*_max_, Vpm, Vmax and the diastolic cardiac parameter −d*p*/d*t*_max_, were all significantly decreased in the rats with MI. By contrast, the diastolic cardiac parameter, LVEDP, was significantly increased. These changes were prevented by treatment with natakalim in a dose-dependent manner. Long-term treatment with natakalim did not affect the HR of the rats in the MI group.

### Natakalim improves LVR induced by MI

**Figure 1:**
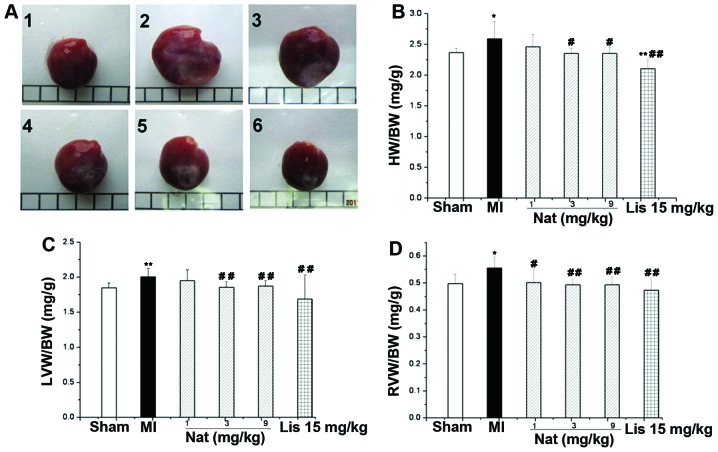
Effects of natakalim on (A) heart weight (HW), (B) HW/body weight (BW) ratio, (C) left ventricular weight (LVW)/BW ratio and (D) right ventricular weight (RVW)/BW ratio in rats with myocardial infarction (MI). (A) Representative macroscopic image of hearts; Panel 1, sham-operated group; panel 2, MI group; panel 3, natakalim 1 mg/kg/day group; panel 4, natakalim 3 mg/kg/day group; panel 5, natakalim 9 mg/kg/day group; panel 6, positive control (lisinopril 15 mg/kg/day). (B-D) Quantification results. Left ventricular hypertrophy was characterized by an increase in the HW/BW, LVW/BW and RVW/BW ratios. These effects were reversed by treatment with natakalim at all doses for 8 weeks. Data are expressed as the means ± SD, n=10–12; ^*^P<0.05 and ^**^P<0.01 vs. sham-operated rats (sham); ^#^P<0.05 and ^##^P<0.01 vs. rats with MI. Nat, natakalim; Lis, lisinopril.

**Figure 2:**
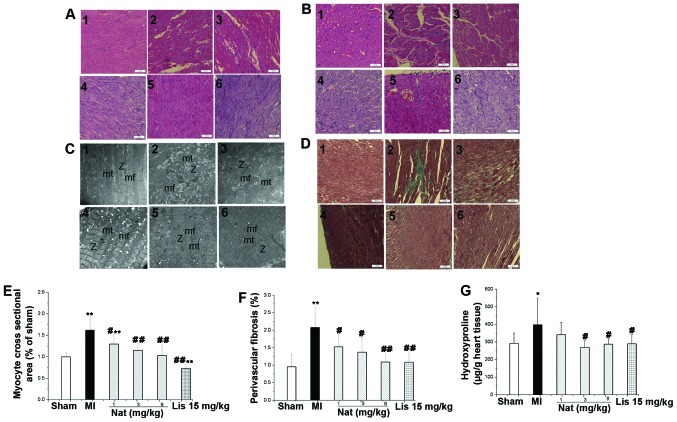
Effects of natakalim on myocyte cross-sectional area, myocardial fibrosis, ultrastructural pathological changes and hydroxyproline content of cardiac tissue in rats with myocardial infarction (MI). (A) Representative myocyte long axis images (H&E staining, ×100 magnification); (B) representative myocyte cross-sectional images (H&E staining, ×200 magnification); (C) representative ultrastructural pathological changes in cardiac tissues (transmission electron microscope micrographs, ×15,000 magnification); (D) representative myocardial fibrosis (Masson’s staining, ×100 magnification); panel 1, sham-operated group; panel 2, MI group; panel 3, natakalim 1 mg/kg/day group; panel 4, natakalim 3 mg/kg/day group; panel 5, natakalim 9 mg/kg/day group; panel 6, positive control (lisinopril 15 mg/kg/day). (E) Quantification of myocyte cross-sectional area; (F) quantification of myocardial fibrosis; (G) quantification of hydroxyproline content in cardiac tissue. Myocyte cross-sectional area, myocardial fibrosis and cardiac hydroxyproline content both increased significantly when compared with the rats with heart failure. Treatment with natakalim at all doses for 8 weeks reversed these pathological changes in left ventricular hypertrophyic parameters. Ultrastructural examination of hearts revealed well-organized myofibrils with mitochondria grouped along the periphery of longitudinally oriented fibers in natakalim group rats. Myocyte cross-sectional area and myocardial fibrosis data are expressed as the means ± SD, n=6; hydroxyproline content data are expressed as the means ± SD, n=11–12; ^*^P<0.05, ^**^P<0.01 vs. sham-operated rats (sham); ^#^P<0.05, ^##^P<0.01 vs. rats with MI. mf, myofibrils; mi, mitochondria; Z, Z-line. Nat, natakalim; Lis, lisinopril.

The results at 8 weeks for all groups are shown in Figs. 1 and 2. LVR in the remote zone was characterized by augmentation of the HW/BW and LVW/BW ratios (Fig. 1B and C), the myocyte cross-sectional area (Fig. 2E) and interstitial myocardial fibrosis (Fig. 2D, E and G). Histological examination (H&E and Mason’s staining) revealed that myocardial tissue damage, compensatory cardiac hypertrophy and myocardial fibrosis of the heart occurred in the left ventricular myocardium (Fig. 2A, B and D). Histological analysis of the hearts of the rats from the MI group indicated that the myocyte cross-sectional area and the extent of myocardial fibrosis were significantly increased compared with the hearts of the rats in the control group (Fig. 2E and F). The hydroxyproline content reflects the amount of collagen in cardiac tissue, and it was increased by 36.4% in the MI group compared with the sham-operated group (Fig. 2G). Ultrastructural examination revealed myofibril disarray, fewer mitochondria and the destruction of mitochondrial membranes in the rats in the MI group (Fig. 2C). Natakalim administered at all doses for 8 weeks reversed these changes.

### Natakalim prevents the progression of hypertrophy to cardiac failure

**Figure 3:**
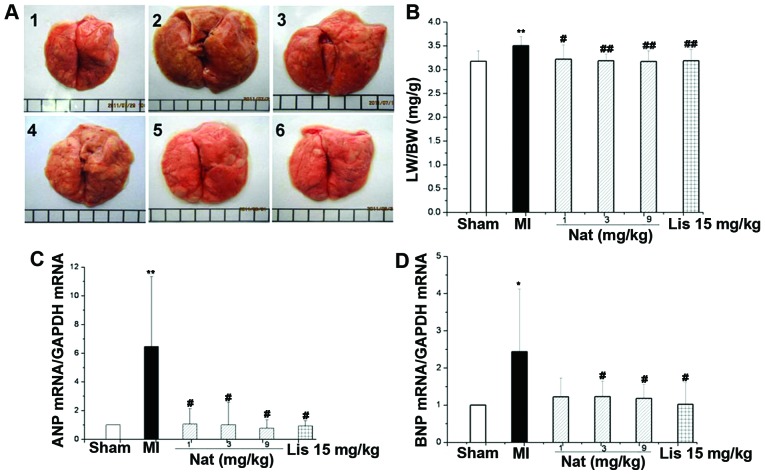
Effects of natakalim on lung index [lung weight (LW)/body weight (BW)] ratio and the expression of atrial natriuretic peptide (ANP) and brain natriuretic peptide (BNP) mRNA in rats with MI. (A) Representative lung macroscopic images; panel 1, sham-operated group; panel 2, MI group; panel 3, natakalim 1 mg/kg/day group; panel 4, natakalim 3 mg/kg/day group; panel 5, natakalim 9 mg/kg/day group; panel 6, positive control (lisinopril 15 mg/kg/day). (B) Quantification of LW/BW ratio; (C and D) quantification of relative expression levels of ANP and BNP mRNA. The progression of left ventricular hypertrophy to heart failure was characterized by an increased LW/BW ratio, the overexpression of ANP and BNP mRNA and increased right ventricular weight (RVW)/BW ratio. The LW/BW and RVW/BW ratios increased and the ANP and BNP mRNA expression was markedly increased in the rats with MI compared with the sham-operated rats. These effects were completely reversed by treatment with natakalim at all doses for 8 weeks. Data are expressed as the means ± SD, n=10–12; ^*^P<0.05 and ^**^P<0.01 vs. sham-operated rats (sham); ^#^P<0.05, ^##^P<0.01 vs. rats with MI. Nat, natakalim; Lis, lisinopril.

The transition from LVR to failure in experimental models is characterized by pulmonary, chronic, right ventricular hypertrophy and the overexpression of ANP and BNP mRNAs, 2 molecular markers of heart failure (11,12). As shown in Figs. 1D and 3, the LW/BW and RVW/BW ratios were increased, and ANP and BNP mRNA expression was markedly increased in the MI group compared with the sham-operated group. These changes were completely reversed by treatment with natakalim at all doses for 8 weeks.

### Effects of natakalim on nitric oxide and NO synthases

**Figure 4:**
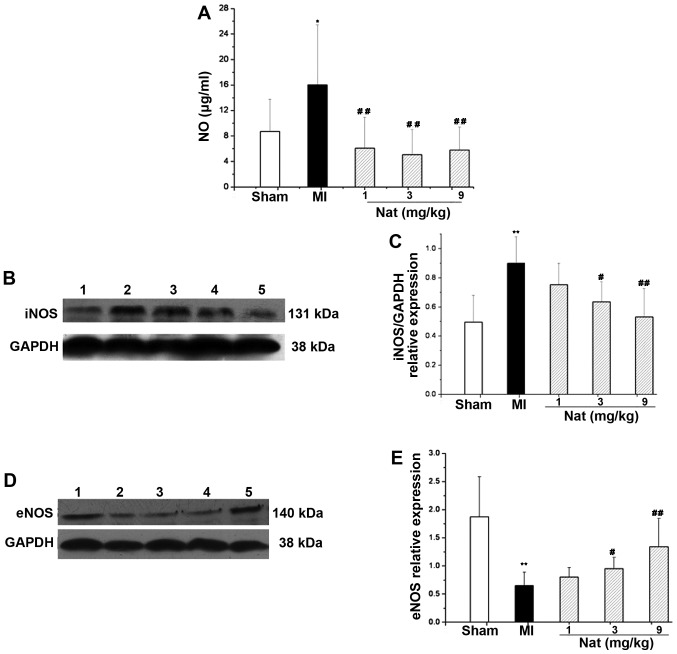
Effects of natakalim on serum concentration of nictic oxide (NO), inducible nitric oxide synthase (iNOS) and endothelial nitric oxide synthase (eNOS) protein expression in rats with myocardial infarction (MI). (A) Quantification of serum content of nitric oxide; (B and D) representative blots of iNOS and eNOS protein expression (western blot analysis); lane 1, sham-operated group; lane 2, MI group; lane 3, natakalim 1 mg/kg/day group; lane 4, natakalim 3 mg/kg/day group; lane 5, natakalim 9 mg/kg/day group; (C and E) quantification of relative values of iNOS and eNOS protein expression. The relative values of eNOS and eNOS protein expression normalized to GAPDH as an internal protein control. Serum NO and iNOS protein expression was increased, whereas eNOS protein expression was decreased in the rats with MI. These effects were reversed by treatment with natakalim at all doses for 8 weeks. Serum NO data are expressed as the means ± SD, n=10–12; eNOS and iNOS data are expressed as the means ± SD, n=6; ^**^P<0.01 vs. sham-operated rats (sham); ^#^P<0.05 and ^##^P<0.01 vs. rats with MI. Nat, natakalim.

eNOS protein expression was significantly reduced in the MI group; however, the serum NO concentration and iNOS protein expression were significantly increased (P<0.01; Fig. 4). Treatment with natakalim for 8 weeks restored the concentration of serum NO to normal levels; the protein expression of eNOS increased to almost normal levels and the protein expression of iNOS in cardiac tissue decreased to almost normal levels.

### Effects of natakalim on the endothelial system

**Figure 5:**
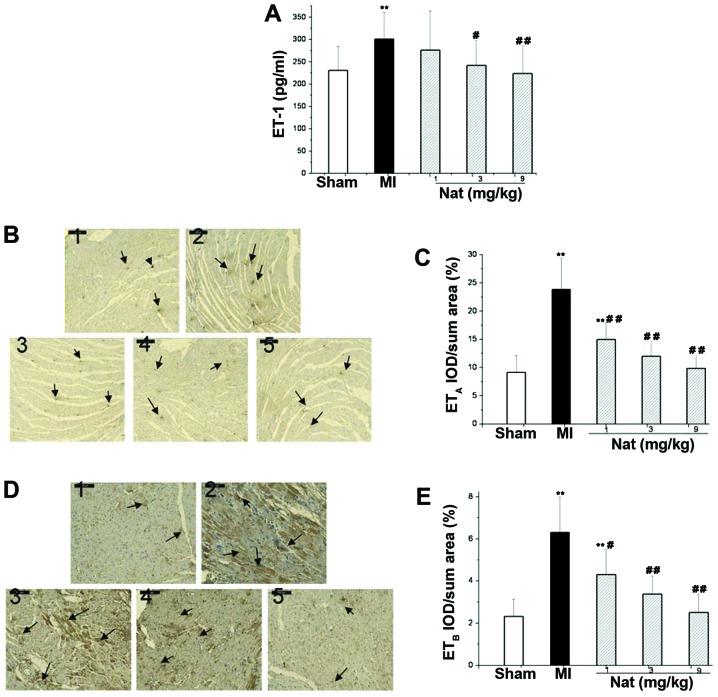
Effects of natakalim on plasma concentration of endothelin (ET)-1 and protein expression of endothelin receptors A and B in cardiac tissue of rats with myocardial infarction (MI). (A) Quantification of plasma concentration of ET-1; (B and D) representative images of ET_A_ and ET_B_ protein expression (immunohistological staining, ×100 magnification); (C and E) quantification of relative values of ET_A_ and ET_B_ protein expression. (B and D) Panel 1, sham-oeprated group; panel 2, MI group; panel 3, natakalim 1 mg/kg/day group; panel 4, natakalim 3 mg/kg/day group; panel 5, natakalim 9 mg/kg/day group. The plasma concentration of ET-1 and the cardiac tissue protein levels of ET_A_ and ET_B_ measured by immunohistochemistry were significantly increased in the rats with heart failure. These effects were reversed by treatment with natakalim at all doses for 8 weeks. Serum ET-1 data are expressed as the means ± SD, n=10–12; ET_A_ and ET_B_ data are expressed as the means ± SD, n=6; ^**^P<0.01 vs. sham-operated rats (sham); ^#^P<0.05 and ^##^P<0.01 vs. rats with MI. Nat, natakalim.

The serum concentration of ET-1 and the cardiac tissue protein levels of ET_A_ and ET_B_ were significantly increased in the rats following MI (P<0.01; Fig. 5). These findings indicate that the MI-induced CHF led to an increase in the synthesis and release of ET-1 and to the increased expression of ET receptors. Treatment with natakalim for 8 weeks markedly attenuated the elevated serum levels of ET-1 and the production of ET_A_ and ET_B_ proteins in the cardiac tissue; the levels returned to almost normal levels.

### Effects of natakalim on the concentration of plasma PGI_2_ and TXA_2_

**Figure 6:**
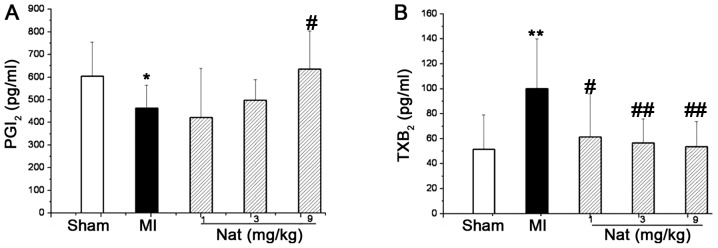
Effects of natakalim on plasma concentration of (A) prostaglandin I2 (PGI_2_) and (B) thromboxane A_2_ (TXA_2_) in rats with myocardial infarction (MI). The concentration of plasma PGI_2_ was reduced, and the concentration of plasma of TXA_2_ was increased in the rats with MI. These effects were reversed by treatment with natakalim at all doses for 8 weeks. Data are expressed as the means ± SD, n=10–12; ^*^P<0.05 and ^**^P<0.01 vs. sham-operated group; ^#^P<0.05 and ^##^P<0.01 vs. MI group. Nat, natakalim.

The plasma levels of prostaglandin F_1a_ (6-keto-PGF_1a_) and thromboxane B_2_ (TXB_2_), metabolites of PGI_2_ and TXA_2_, respectively, were used to estimate the plasma levels of PGI_2_ and TXA_2_, respectively, due to their short half-lives. In the rats with CHF induced by MI, the concentration of plasma 6-keto-PGF_1a_ was significantly decreased (Fig. 6A), while the concentration of plasma TXB_2_ was significantly increased (Fig. 6B). Treatment with natakalim for 8 weeks restored the concentrations of plasma 6-keto-PGF_1a_ and TXB_2_ to almost normal levels.

## Discussion

To the best of our knowledge, the present study is the first to evaluate the effects of natakalim, a novel SUR2B/Kir6.1 channel opener, on post-infarction LVR and CHF in rats. The major findings of the present study include the following observations: i) natakalim reverses pathological LVR in a rat model of LAD ligation; ii) natakalim prevents the progression from hypertrophy to CHF; iii) the underlying mechanisms of action of natakalim may be attributed to the restoration of a coordinated balance between endothelial function and cardiac hypertrophy by the correction of endothelial dysfunction, the decrease in NO and the increase in PGI_2_ secretion, the inhibition of ET-1 and TXA_2_ biosynthesis and secretion, the increase in cardiac tissue eNOS protein expression, and the decrease in cardiac tissue iNOS, ET_A_ and ET_B_ receptor protein expression.

CHF is caused by multiple factros that result from the heart being unable to pump sufficient blood to meet the metabolic requirements of the body. The heart undergoes a compensatory remodeling process in response to initial myocardial insults. The remodeling initially helps the heart to preserve pump function; however, the heart ultimately becomes maladaptive and fails to function. Ischemic heart disease, the most important etiology of CHF, accounts for up to two-thirds of all CHF cases. Hypertension is another important factor; thus, the rat model of pressure overload that leads to the development of concentric hypertrophy and systolic dysfunction is widely used to simulate the progression of hypertensive heart disease (16). We have previously demonstrated that natakalim prevents LVR and the progression from hypertrophy to failure induced by pressure overload (12). Therefore, in this study, we explored the effects of natakalim on LVR and heart failure in a rat model of MI.

The LAD ligation model involves the MI-induced progression from hypertrophy to cardiac failure and has been characterized extensively (15–18). In the present study, we found that following LAD ligation, the infarcted wall of the heart was gradually replaced by scar tissue, and the non-infarcted myocardium exhibited LVR, including cardiac hypertrophy, interstitial myocardial fibrosis and changes in left ventricular shape; these findings are consistent with those of previous studies (15–18). The hypertrophy of the non-infarcted myocardium, which may not appear to be damaged at the time of the infarction, cannot compensate sufficiently to prevent the progression of heart failure, and this inability to compensate is identical to our findings, such as pulmonary congestion, right ventricular hypertrophy, overexpression of the heart failure indicators, ANP and BNP mRNA, and the depression of cardiac systolic and diastolic function (11,12). To the best of our knowledge, the present study is the first to evaluate the effects of natakalim on LVR and CHF induced by MI. Following treatment with natakalim for 8 weeks, the LVW/BW ratio, cardiomyocyte cross-sectional area, the extent of interstitial myocardial fibrosis, hydroxyproline content and pathological damage in cardiac tissue were all reversed, and the progression from LVR to failure was prevented. These results suggest that natakalim can prevent LVR and the progression from hypertrophy to failure induced by MI. Taken together, these findings suggest that natakalim may be a suitable therapy for patients with post-infarction CHF.

However, the mechanisms responsible for the progression of LVR to heart failure remain poorly understood. An increasing body of evidence has drawn attention to the role of the coronary endothelium (6,19,20). Coronary endothelial dysfunction is related to the development of myocardial ischemia, impairment in contractility and the progression to CHF (6). Furthermore, it has been reported that the imbalance between endothelial function and the adaptation of cardiac hypertrophy causes the transition of LVR to heart failure (7). Therefore, a novel therapeutic strategy for post-MI heart failure may be to correct coronary endothelial dysfunction in the non-infarcted myocardium and restore the coordinated balance between endothelial function and cardiac hypertrophy.

The vascular endothelium produces and releases a wide range of vasoactive factors to regulate vascular homeostasis, such as NO, ET-1, angiotensin II (AngII), PGI_2_ and TXA_2_ (3,4,19,20). Endothelial dysfunction is an important cardiovascular risk factor, and it may result from a variety of factors, including augmented ET-1 synthesis and reduced endothelium-derived NO synthesis. Endothelial dysfunction may also result from the imbalance between the formation and release of PGI_2_ and TXA_2_ into the circulation. A growing body of evidence suggests that NO, ET-1 and PGI_2_ are associated with cardiac hypertrophy and heart failure following MI (20–22).

Previous studies have suggested that LVR and the development of failure are regulated by eNOS, iNOS and NO. At a low concentration, NO synthesized by eNOS in the endotheliocyte contributes to the maintenance of vascular tone and function, protects against myocyte hypertrophy and apoptosis and preserves cardiomyocyte contractility (5). Compared with wild-type mice, eNOS knockout mice display significantly aggravated LVR and impaired myocardial function following MI (23), whereas eNOS overexpression improves survival and cardiac function following MI (24). In the normal adult heart, iNOS has not been found to be constitutively present, but iNOS can be induced by ischemia, heart failure, stroke and infection (25). iNOS may lead to the production of large amounts of NO for a sustained period of time, but the excessive amounts of NO are sufficiently cytotoxic, inducing the apoptosis of cardiomyocytes and impairing cardiac contractile function, which has been demonstrated in patients with CHF and animal models (26,27). The protective effects exerted by natakalim against post-MI CHF are related to the reduction in iNOS-derived NO and the increase in eNOS-derived NO. Further evidence indicates that inflammation is involved in post-MI CHF and that iNOS and eNOS participate in the inflammation of CHF (28). Iptakalim can suppress the inflammatory reaction by counteracting endothelial dysfunction (29); however, determining whether the protective effects exerted by its derivative, natakalim, involve the suppression of inflammation in post-MI CHF requires further investigation.

The endothelin system, particularly ET-1 and the ET_A_ and ET_B_ receptors, has been implicated in pathological states, such as ventricular remodeling and CHF following MI (30). ET-1 is the most potent endothelium-derived vasoconstrictor peptide, and it can stimulate myocardial hypertrophy, myocardial fibrosis and cardiomyocyte injury. The actions of ET-1 are mediated through the activation of ET_A_ and ET_B_ receptors, which are found in a variety of cells of the cardiovascular system (31). Elevated circulating ET-1 levels are observed in the failing hearts of patients and animals with CHF, whereas suppressing circulating ET-1 levels during the acute phase of MI prevents post-MI LVR in patients (30). Moreover, ET_A_ and ET_B_ receptor antagonists prevent progressive LVR and improve cardiac function in rats with a large MI (30,32,33). Taken together, this evidence suggests that the downregulation of the endothelin system alleviates cardiomyocyte hypertrophy and prevents LVR and CHF following acute MI.

PGI_2_ and TXA_2_ are active metabolites of arachidonic acid and PGI_2_ is thought to be a physiological antagonist of TXA_2_. PGI_2_ is generated by vascular endothelial cells, which possess strong vasodilator, anti-aggregatory and antihypertrophic effects. TXA_2_ is generated by activated platelets and neutrophilic granulocytes when vascular endothelial cells are injured, and TXA_2_ exhibits pro-aggregatory, vasoconstrictor properties and stimulates vascular remodeling (34). PGI_2_ receptor IP knockout mice develop significant LVR and severe myocardial fibrosis following MI, aortic banding or salt-sensitive hypertension (35–37). Thus, an imbalance between PGI_2_ and TXA_2_ is deleterious for the circulatory system and can lead to vascular diseases, such as atherosclerosis, MI, post-MI LVR and hypertension.

K_ATP_ channels are membrane sensors of energy metabolism, and play an important role in a number of diseases, such as MI and CHF. It has been reported that the SUR2B/Kir6.1 subtype is mainly found in the endothelium, and it affects the production and release of endothelial autacoids, such as NO, PGI_2_ and ET-1 (38). A series of studies carried out in our laboratory have suggested that natakalim has a high selectivity for the SUR2B/Kir6.1 subtype of K_ATP_ channels in endotheliocytes and promotes the secretion of NO and PGI_2_ and inhibits the production of ET-1 in endotheliocytes (12–14,39). In our rat model of post-MI heart failure, natakalim increased eNOS protein expression and the serum concentration of endothelium-derived NO, reduced iNOS protein expression, and attenuated the pathologically abnormal increase in NO concentration, all of which counteracted the deregulation of the endothelium-derived NO system. In addition, natakalim reduced plasma ET-1 levels and protein expression of ET_A_ and ET_B_ receptors, suppressing the endothelin system. Additionally, natakalim increased the plasma PGI_2_ concentration and decreased the plasma TXA_2_ concentration, which corrected the imbalance between PGI_2_ and TXA_2_. Therefore, as previously noted, natakalim can correct coronary endothelial dysfunction in the non-infarcted myocardium following post-MI heart failure.

In conclusion, to the best of our knowledge, the present study demonstrates for the first time that natakalim counteracts cardiac hypertrophy and prevents the progression to heart failure induced by MI. The underlying molecular mechanisms involved the restoration of the coordinated balance between endothelial function and cardiac hypertrophy by correcting endothelial dysfunction, including the amelioration of the endothelium-derived NO system, the inhibition of the endothelin system and the rectification of the imbalance between PGI_2_ and TXA_2_. Thus, natakalim may provide a novel therapeutic strategy for correcting endothelial dysfunction. Although natakalim appears to be suitable for the clinical treatment of coronary heart disease and post-infarction heart failure, a series of well-designed trials is required in order to confirm our results.

## Figures and Tables

**Table I:** Effects of natakalim on HR, MABP and hemodynamic parameters in the rats with myocardial infarction (MI).

Group	n	HR/min	MABP (kPa)	LVSP (kPa)	+d*p*/d*t*_max_ (kPa/s)	Vpm(/sec)	Vmax(/sec)	LVEDP (kPa)	−d*p*/d*t*_max_ (kPa/sec)
Sham-operated	11	405±21	14.69±1.07	20.10±0.81	440.0±48.0	0.919±0.100	0.542±0.176	6.04 ±0.48	409.4±57.9
MI	12	388±34	13.75±1.20^a^	19.03±0.70^a^	382.6±51.9^a^	0.753±0.120^b^	0.319±0.186^b^	7.23±0.81^b^	346.0±61.6^a^
Nat 1 mg/kg/day	11	399±29	14.98±1.41^d^	19.82±0.72^d^	438.2±49.2^d^	0.890±0.116^d^	0.444±0.192	6.41±0.54^d^	407.2±51.2^c^
Nat 3 mg/kg/day	12	393±33	15.08±0.89^d^	19.88±0.33^c^	431.5±28.0^d^	0.899±0.084^d^	0.530±0.168^d^	6.26±0.54^d^	403.3±34.4^c^
Nat 9 mg/kg/day	12	406±30	15.17±0.70^d^	19.78±0.78^c^	455.0±34.0^d^	0.977±0.076^d^	0.639±0.156^d^	5.82±0.34^d^	433.6±44.2^d^
Lis 15 mg/kg/day	11	413±31	12.16±1.15^b,d^	18.64±0.55^b^	366.7±25.9^b^	0.724±0.058^b^	0.214±0.108^b^	7.40±0.30^b^	345.4±26.8^b^

Data are presented as the means ± SD; ^a^P<0.05 and ^b^P<0.01 vs. sham-operated group; ^c^P<0.05 nd ^d^P<0.01 vs. MI group. +d*p*/d*t*_max_, maximal rate of rise in left ventricular pressure; −d*p*/d*t*_max_, maximal rate of decrease in left ventricular pressure; HR, heart rate; MABP, mean arterial blood pressure; LVSP, left ventricular systolic pressure; LVEDP, left ventricular end diastolic pressure; Vmax, the maximal velocity of contractile element shorting; Vpm, the physiological velocity of contractile element shorting; Nat, natakalim (1, 3 and 9 mg/kg/day orally for 8 weeks); Lis, lisinopril (15 mg/kg/day orally for 8 weeks).
